# NIH: THE SIMPLIFYING REVIEW FRAMEWORK

**DOI:** 10.1093/geroni/igae098.1801

**Published:** 2024-12-31

**Authors:** Elia Ortenberg

**Affiliations:** National Institutes of Health, Bethesda, Maryland, United States

## Abstract

This presentation will discuss NIH’s current initiative, which will be implemented for applications submitted after January 25, 2025, to simplify the peer review framework for most research project grant applications. NIH’s ultimate goal through this effort is to identify the best, most innovative science with the potential to improve human health or advance our scientific understanding. The initiative involves reorganizing the existing review criteria in a way that will help reviewers focus on the key questions needed to assess scientific merit of applications: Can and should the proposed research be conducted? We will present a brief history that drove the changes and provide an overview of the new review criteria as well as the impact on the scoring method and arrangement of the summary statement that applicants will receive.

